# Supplementary material for: Plasma microRNAs Associate Positive, Negative, and Cognitive Symptoms with Inflammation in Schizophrenia
Source: Int J Mol Sci. 2024 Dec 17;25(24):13522. doi: 10.3390/ijms252413522 (PMC11676741; doi:10.3390/ijms252413522)
Supplement: Supplementary file 1 [file ijms-25-13522-s001.zip › Supplemental Figures.pdf]

Supplimentary Figures

Plasma microRNAs associate positive, negative, and cognitive symptoms with inflammation in schizophrenia

Takuya Miyano, Masakazu Hirouchi, Naoki Yoshimura, Kotaro Hattori, Tsuyoshi Mikkaichi, and Naoki Kiyosawa

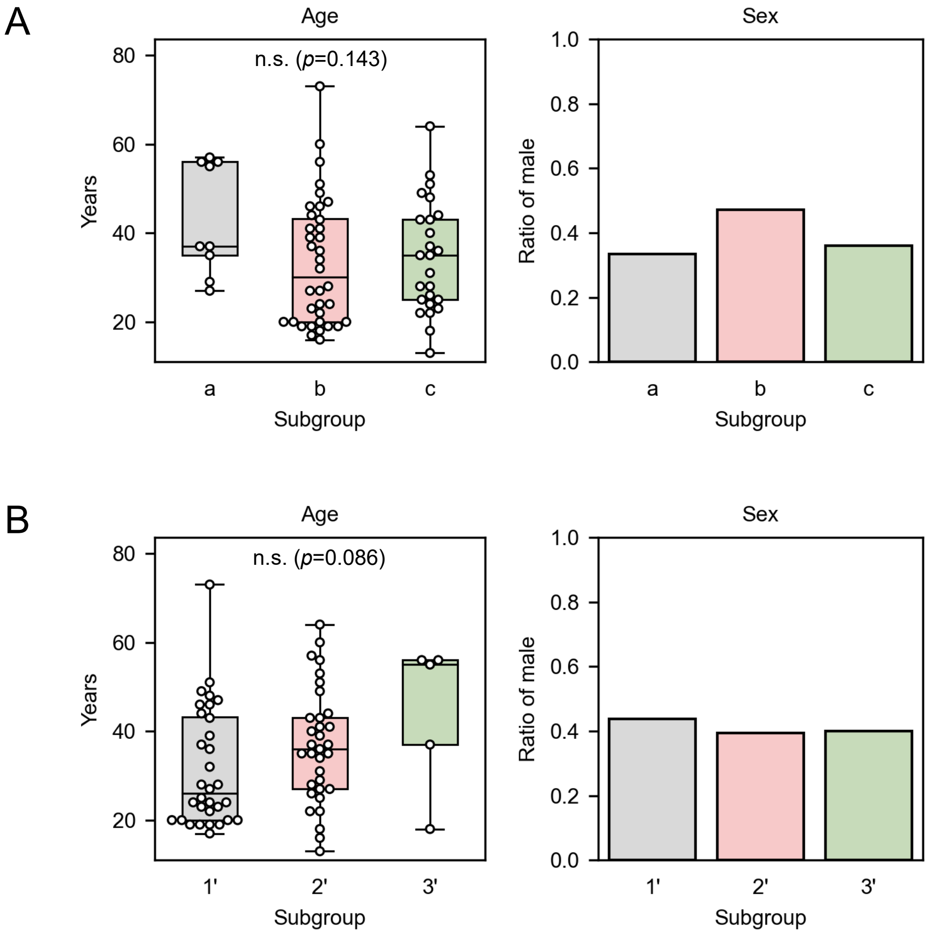

Figure S1. Age and sex were not different among the miRNA-based subgroups. Box-and-swarm plots show patient's age in each subgroup. Bar plots show ratio of male in each subgroup. A one-way analysis of variance was conducted to examine the effect of subgroups on age. **(A)** miRNA-based subgroups *a*, *b*, and *c* based on overall miRNA profiles (B) miRNA-based subgroups 1´, 2´, and 3´ based on the pre-defined 40 miRNAs.

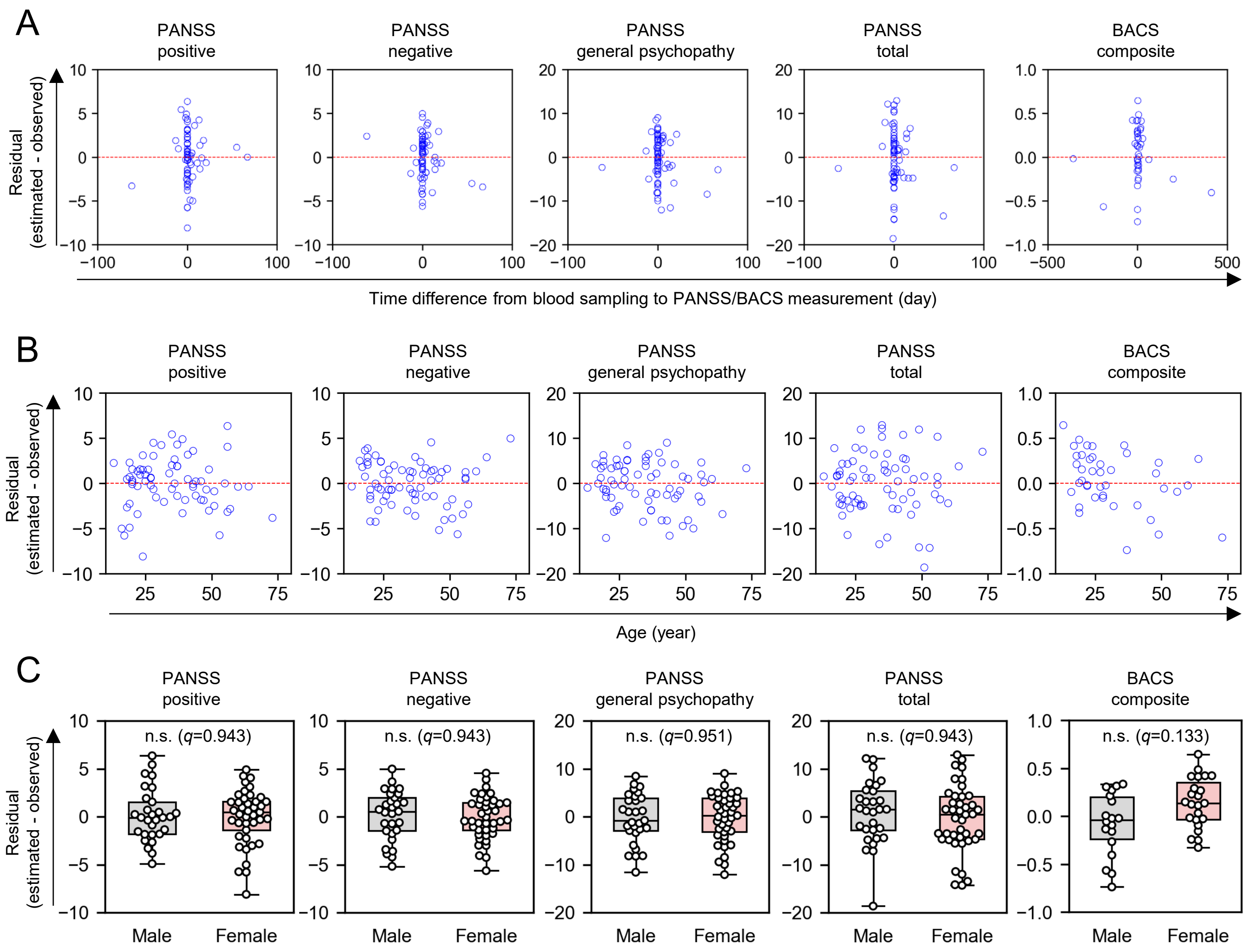

Figure S2. Time difference from blood sampling to PANSS/BACS measurement, age, and sex had no influence on the estimation accuracy. Scatter plots show the relationship between time difference from blood sampling to PANSS/BACS measurement **(A)** or age **(B)** and the residual (i.e., the estimated symptom score minus the observed symptom score for each sample) in the leave-one-out cross validation. The red dashed line represent the line of perfect agreement between observed and estimated symptom scores. **(C)** Box-and-swarm plots show the residual in the leave-one-out cross validation in each sex. *t*-test was applied to compare the residuals between the sex. q denotes Benjamini–Hochberg corrected p value.
